# Hyperlactatemia and altered lactate kinetics are associated with excess mortality in sepsis: A multicenter retrospective observational study

**DOI:** 10.1007/s00508-022-02130-y

**Published:** 2022-12-28

**Authors:** Richard Rezar, Behrooz Mamandipoor, Clemens Seelmaier, Christian Jung, Michael Lichtenauer, Uta C. Hoppe, Reinhard Kaufmann, Venet Osmani, Bernhard Wernly

**Affiliations:** 1 Department of Cardiology and Intensive Care Medicine, Paracelsus Medical University of Salzburg, 5020 Salzburg, Austria; 2 Fondazione Bruno Kessler Research Institute, Trento, Italy; 3 Division of Cardiology, Pulmonology and Vascular Medicine, Medical Faculty, University of Düsseldorf, Düsseldorf, Germany; 4 Department of Radiology, Paracelsus Medical University of Salzburg, Salzburg, Austria; 5 Information School, University of Sheffield, Sheffield, UK; 6 Department of Internal Medicine, General Hospital of Oberndorf, Oberndorf, Austria; 7 Center for Public Health and Healthcare Research, Paracelsus Medical University of Salzburg, Salzburg, Austria

**Keywords:** Biomarkers, Risk assessment, Prognosis, Clinical decision-making, Critical care outcomes

## Abstract

Severe hyperlactatemia (>10mmol/L) or impaired lactate metabolism are known to correlate with increased mortality. The maximum lactate concentration on day 1 of 10,724 septic patients from the eICU Collaborative Research Database was analyzed and patients were divided into three groups based on maximum lactate in the first 24 h (<5mmol/l; ≥5mmol/l & <10mmol/l; ≥10mmol/l). In addition, delta lactate was calculated using the following formula: (maximum lactate day 1 minus maximum lactate day 2) divided by maximum lactate day 1. A multilevel regression analysis was performed, with hospital mortality serving as the primary study end point. Significant differences in hospital mortality were found in patients with hyperlactatemia (lactate ≥10mmol/l: 79%, ≥5mmol/l & <10mmol/l: 43%, <5mmol/l, 13%; p<0.001). The sensitivity of severe hyperlactatemia (≥10mmol/l) for hospital mortality was 17%, the specificity was 99%. In patients with negative delta lactate in the first 24 h, hospital mortality was excessive (92%). In conclusion, mortality in patients with severe hyperlactatemia is very high, especially if it persists for more than 24 h. Severe hyperlactatemia, together with clinical parameters, could therefore provide a basis for setting treatment limits.

## Introduction

According to the “Sepsis-3” definition, sepsis is a “life-threatening organ dysfunction caused by a dysregulated host response to infection” [1]. Furthermore, if inadequate tissue oxygen utilization occurs, this is referred to as “shock”, which is usually accompanied by an increasingly poor prognosis [1, 2]. As sepsis is one of the most common causes of death in intensive care units (ICUs) worldwide, and despite ongoing medical developments a high mortality and morbidity with often limiting long-term sequelae can be observed, methods for deciding who benefits from intensive treatment are desired [3–5].

Determination of serum lactate has played a relevant role in intensive care medicine for years and is a relevant parameter in sepsis and (septic) shock [1, 2]. This was noticed as early as 1943, when Engel et al. in their work on hemorrhagic shock in a rat model, described “a lactate rise and pyruvate elevation that appears to be related to the effects of decreased oxygenation of peripheral tissues and the liver” [6]. Lactate was often considered an indicator of hypoxia at the cellular level, but this concept is no longer generally accepted. Meanwhile it is known that lactate is also produced independently of hypoxia as part of an increased cellular metabolism via enhanced glycolysis [7]. Nevertheless, it can certainly be considered as an indicator of a state of alarm at the tissue level. The role of lactate in prognostication and risk stratification especially in critically ill patients is well established and largely undisputed [7–9]. Lactate measurements are readily available in ICUs worldwide by point-of-care testing (POCT) devices. Due to its inexpensive and ubiquitous availability, in addition to its multiply demonstrated diagnostic value, the parameter is well suited for follow-up and comparative purposes; however, not only its short-term and medium-term course but also the absolute value of initial measures make it an interesting and important tool in risk stratification.

Particularly severe hyperlactatemia is a robust surrogate parameter for critical illness and high mortality and can be interpreted from two points of view [8]. On the one hand, the presence of severe hyperlactatemia should trigger a thorough clinical evaluation of the affected patient to detect the etiology of the critical illness and initiate possible targeted therapy. On the other hand, it should also be used as an opportunity to re-evaluate the treatment goal in the context of other clinical findings.

We analyzed the incidence of severe hyperlactatemia and the prognosis of affected individuals in a large cohort of septic patients in the multicenter eICU Collaborative Research Database to shed light on this particular subgroup of seriously ill individuals. With this study, we aimed to better define the different subgroups of septic patients with hyperlactatemia, as well as to better characterize survivors and non-survivors from the subgroup of patients with severe hyperlactatemia and patients with or without positive lactate kinetics. Additionally, the diagnostic value of severe hyperlactatemia was calculated. We hypothesized that patients with sepsis and severe hyperlactatemia, as well as individuals without positive 24‑h lactate kinetics, would have a significantly worse outcome.

## Patients, material and methods

### Study subjects

Critically ill patients admitted to an intensive care unit (ICU) diagnosed with sepsis according to Acute Physiology and Chronic Health Evaluation (APACHE) IV and a documented lactate concentration on day 1 were included in this analysis from the eICU Collaborative Research Database [9]. Septic shock was defined as a diagnosis of sepsis by means of APACHE IV, vasopressor requirement and serum lactate level greater than 2 mmol/L. The eICU database is a multicenter intensive care unit database, which includes over 200,000 admissions in 335 ICUs across the USA in 2014 and 2015 and is released under the Health Insurance Portability and Accountability Act (HIPAA) safe harbor provision (Privacert, Cambridge, MA, USA; HIPAA Certification no. 1031219‑2; [9, 10]). An institutional review board (IRB; Massachusetts Institute of Technology, Cambridge, MA, USA) approval exists.

### Statistical analysis

We expressed continuous data points as median ± interquartile range (IQR) and differences between independent groups using Kruskal-Wallis equality of populations rank test accordingly. Categorical data are given as numbers (percentage), the χ^2^-test was used to calculate univariate differences between groups. The primary exposure was hyperlactatemia on day 1 categorized into low (group 1; maximum lactate concentration < 5 mmol/L), high (group 2; maximum lactate concentration ≥ 5 and < 10 mmol/L) and severe hyperlactatemia (group 3; maximum lactate concentration ≥ 10 mmol/L). Delta-lactate was calculated by the following formula: (maximum lactate on day 1 minus maximum lactate on day 2) divided by maximum lactate on day 1. The primary outcome of our analysis was hospital mortality. We evaluated the frequencies of mechanical ventilation (MV), vasopressor use, acute kidney injury (AKI) within 48 h based on serum creatinine and according to the Kidney Disease Improving Global Outcomes (KDIGO) guidelines, length of stay (LOS) and ICU mortality as secondary outcomes [11]. To correct for missing data list-wise deletion was applied. To adjust for potential confounders, we used a multilevel logistic regression analysis to fit two models: [1] a baseline model with the hospital as a random effect and lactate concentration as fixed effect, as well as [2] a multivariable model adjusting for APACHE IV. We calculated adjusted odds ratios (aOR) with respective 95% confidence intervals (95% CI). We calculated sensitivity, specificity, positive and negative likelihood ratios (LR+ and LR−) for severe hyperlactatemia. All tests were two-sided, and a *p*-value of < 0.05 was considered statistically significant. We used Stata/IC 16.1 (StataCorp. 2019. Stata Statistical Software: Release 16. College Station, TX, USA: StataCorp LLC) for all statistical analyses.

## Results

### General characteristics

A total of 10,724 patients were included in the analysis, whereas 9208 patients had a maximum lactate < 5 mmol/l (group 1), 1056 patients were in the high lactate group (group 2; lactate ≥ 5 and < 10 mmol/L) and 460 patients had severe hyperlactatemia (group 3; lactate ≥ 10 mmol/L). Patients in group 3 were younger (median age 65 years, IQR 56–75 years) compared with patients in group 2 (67, IQR 56–79 yrs.) and group 1 (67, IQR 56–78 yrs.; *p* = 0.041), but no difference was found between male and female patients (male sex; groups 1–3: 51% vs. 52% vs. 48%, respectively; *p* = 0.39). As for ethnicity, more individuals in group 3 were African American (groups 3‑1 in descending order: 12 vs. 11 vs. 9%; *p* = 0.030), and fewer patients were Caucasian (groups 1–3: 80 vs. 75 vs. 75%; *p* = 0.030). Individuals with severely elevated lactate values showed higher sequential organ failure assessment (SOFA) scores; (groups 3‑1 in descending order: 12 (IQR 9–14) vs. 10 (IQR 7–12) vs. 6 (IQR 3–8) points; *p* < 0.001), as well as APACHE IV (groups 3‑1: 113 (IQR 93–132) vs. 90 (IQR 72–111) vs. 66 (IQR 51–82) points; *p* < 0.001) and suffered from septic shock more often (groups 3‑1 in descending order: 68 vs. 52 vs. 13%; *p* < 0.001). Patients with severe hyperlactatemia had a gastrointestinal (groups 3‑1 in descending order: 24 vs. 20 vs. 13%; *p* < 0.001) or unknown focus of infection (groups 3‑1 in descending order: 18 vs. 13 vs. 11%; *p* < 0.001) more frequently than individuals in the other groups. Also, significant differences were observed for hospital mortality (groups 3‑1 in descending order: 79 vs. 43 vs. 13%; *p* < 0.001), as well as all secondary endpoints (groups 3‑1 in descending order: ICU mortality: 70 vs. 32 vs. 8%, mechanical ventilation: 59 vs. 41 vs. 22%; vasopressor use: 69 vs. 54 vs. 33%; AKI: 22 vs. 14 vs. 6%; ICU LOS: 30 vs. 68 vs. 59 h; all *p* < 0.001). A detailed overview is given in Table 1.

**Table 1:** Differencesbetween patients with low, high and severe hyperlactatemia

Characteristic	Maximum lactate (< 5 mmol/l)	Maximum lactate (≥ 5 and < 10 mmol/l)	Maximum lactate ≥ 10 mmol/l	*p*-value
	*n* *=* *9208*	*n* *=* *1056*	*n* *=* *460*	
Age (years)—median (IQR)	67 (56–78)	67 (56–79)	65 (56–75)	0.041*
Age > 65 years—*n* (%)	4902 (53)	587 (56)	223 (48)	0.038*
Male sex—*n* (%)	4685 (51)	550 (52)	222 (48)	0.39
BMI (kg/m^2^)—median (IQR)	27 (23–33)	26 (22–32)	27 (23–34)	0.001*
SOFA score (pts.)—median (IQR)	6 (3–8)	10 (7–12)	12 (9–14)	< 0.001*
APACHE IV (pts.)—median (IQR)	66 (51–82)	90 (72–111)	113 (93–132)	< 0.001*
Septic shock—*n* (%)	1145 (13)	552 (52)	313 (68)	< 0.001*
*Ethnicity*	–	–	–	0.030*
African American—*n* (%)	860 (9)	121 (11)	55 (12)	–
Asian—*n* (%)	146 (2)	18 (2)	10 (2)	–
Caucasian—*n* (%)	7316 (80)	795 (75)	341 (75)	–
Hispanic—*n* (%)	319 (3)	48 (5)	21 (5)	–
Native American—*n* (%)	89 (1)	8 (1)	6 (1)	–
Other/unknown—*n* (%)	432 (5)	63 (6)	21 (5)	–
*Laboratory values*	–	–	–	–
Maximum lactate day 1 (mmol/L)—median (IQR)	1.7 (1.1–2.6)	6.5 (5.6–7.8)	13.5 (11.5–16.8)	< 0.001*
Baseline lactate > 2 mmol/L—*n* (%)	3193 (35)	1032 (98)	454 (99)	< 0.001*
Hemoglobin (g/dL)—median (IQR)	10.3 (8.9–11.8)	10.5 (8.8–12.1)	9.7 (8.1–11.4)	< 0.001*
White blood count (G/L)—median (IQR)	13.3 (8.8–19.0)	14.1 (7.5–22.3)	14.1 (7.4–22.8)	0.063
Lymphocyte count (G/L)—median (IQR)	0.7 (0.4–1.2)	0.6 (0.3–1.1)	0.8 (0.4–1.3)	< 0.001*
Platelet count (G/L)—median (IQR)	183 (127–253)	150.0 (86–218)	137.0 (71–221)	< 0.001*
Serum creatinine (mg/dL)—median (IQR)	1.3 (0.9–2.3)	2.1 (1.4–3.1)	2.8 (1.9–3.9)	< 0.001*
*Admission diagnosis*	–	–	–	< 0.001*
Pulmonary focus—*n* (%)	3462 (38)	363 (34)	130 (28)	–
GI focus—*n* (%)	1173 (13)	214 (20)	109 (24)	–
Renal focus/UTI—*n* (%)	2172 (24)	205 (19)	71 (15)	–
Gynecological focus—*n* (%)	34 (0)	4 (0)	0 (0)	–
Cutaneous/soft tissue focus—*n* (%)	763 (8)	59 (6)	25 (5)	–
Other focus—*n* (%)	563 (6)	69 (7)	40 (9)	–
Unknown focus—*n* (%)	1041 (11)	142 (13)	85 (18)	–
*Endpoints*	–	–	–	–
Mechanical ventilation—*n* (%)	2052 (22)	434 (41)	271 (59)	< 0.001*
Vasopressor use—*n* (%)	2993 (33)	565 (54)	317 (69)	< 0.001*
AKI—*n* (%)	464 (6)	107 (14)	47 (22)	< 0.001*
LOS—median (IQR)	59 (34–111)	68 (30–141)	30 (18–80)	< 0.001*
LOS > 7 days—*n* (%)	1288 (14)	213 (20)	63 (14)	< 0.001*
ICU mortality—*n* (%)	700 (8)	339 (32)	321 (70)	< 0.001*
Hospital mortality—*n* (%)	1229 (13)	457 (43)	362 (79)	< 0.001*

*statistically significant

*AKI* acute kidney injury, *APACHE IV* Acute Physiology and Chronic Health Evaluation, *BMI* body mass index, *GI* gastrointestinal, *ICU* intensive care unit, *IQR* interquartile range, *LOS* length of stay, *pts.* points, *SOFA* sequential organ failure assessment, *UTI* urinary tract infection

### Survivors versus non-survivors in severe hyperlactatemia

Survivors in the severe hyperlactatemia group were younger compared to non-survivors (60 (IQR 50–72) vs. 66 yrs. (IQR 58–75), *p* < 0.001), and had lower APACHE IV- (98 vs. 116 points, *p* < 0.001) but not SOFA-scores (both 12 points, *p* = 0.22). No difference was shown for focus of infection (*p* = 0.14) or ethnicity (*p* = 0.92). Regarding secondary endpoints, survivors had significantly longer ICU stays (117 vs. 26 hrs.; *p* < 0.001), for all other endpoints (mechanical ventilation: 57 vs. 59%, *p* = 0.69; vasopressor use: 64 vs. 70%, *p* = 0.26; AKI 16 vs. 25%, *p* = 0.091) no significant difference was observed. A detailed overview is given in Table 2.

**Table 2:** Differencesbetween survivors and non-survivors in patients with severe hyperlactatemia

Characteristic	Survivors	Deceased	*p*-value
	*n* *=* *98*	*n* *=* *362*	
Age (years)—median (IQR)	60 (50–72)	66 (58–75)	< 0.001*
Age > 65 years—*n* (%)	39 (40)	184 (51)	0.053
Male sex—*n* (%)	43 (44)	179 (49)	0.33
BMI (kg/m^2^)—median (IQR)	28 (23–32)	27 (23–34)	0.87
SOFA score (pts.)—median (IQR)	12 (9–14)	12 (10–14)	0.22
APACHE IV score (pts.)—median (IQR)	98 (81–122)	116 (97–137)	< 0.001*
Septic shock—*n* (%)	61 (63)	252 (70)	0.19
*Ethnicity*	–	–	0.92
African American—*n* (%)	14 (14)	41 (11)	–
Asian—*n* (%)	3 (3)	7 (2)	–
Caucasian—*n* (%)	69 (71)	272 (76)	–
Hispanic—*n* (%)	5 (5)	16 (4)	–
Native American—*n* (%)	1 (1)	5 (1)	–
Other/Unknown—*n* (%)	5 (5)	16 (4)	–
*Laboratory values*	–	–	–
Maximum lactate day 1 (mmol/L)—median (IQR)	12.4 (10.7–14.6)	13.8 (11.7–17.5)	< 0.001*
Baseline lactate > 2 mmol/L—*n* (%)	96 (98)	358 (99)	0.47
Hemoglobin (g/dL)—median (IQR)	10.2 (8.8–11.8)	9.6 (8.0–11.3)	0.052
White blood count (G/L)—median (IQR)	14.1 (8.8–24.5)	14.2 (6.6–22.5)	0.095
Lymphocyte count (G/L)—median (IQR)	0.8 (0.6–1.0)	0.8 (0.4–1.4)	0.84
Platelet count (G/L)—median (IQR)	156 (85–247)	128 (67–211)	0.020*
Serum creatinine (mg/dL)—median (IQR)	2.6 (1.8–4.2)	2.8 (2.0–3.9)	0.46
*Admission diagnosis*	–	–	0.14
Pulmonary focus—*n* (%)	24 (24)	106 (29)	–
GI focus—*n* (%)	27 (28)	82 (23)	–
Renal focus/UTI—*n* (%)	16 (16)	55 (15)	–
Cutaneous/soft tissue focus—*n* (%)	3 (3)	22 (6)	–
Other focus—*n* (%)	4 (4)	36 (10)	–
Unknown focus—*n* (%)	24 (24)	61 (17)	–
*Endpoints*	–	–	–
Mechanical ventilation—*n* (%)	56 (57)	215 (59)	0.69
Vasopressor use—*n* (%)	63 (64)	254 (70)	0.26
AKI—*n* (%)	13 (16)	34 (25)	0.091
LOS (hours)—median (IQR)	117 (66–221)	26 (16–44)	< 0.001*
LOS > 7 days—*n* (%)	36 (37)	27 (7)	< 0.001*
ICU mortality—*n* (%)	0 (0)	321 (89)	< 0.001*
Hospital mortality—*n* (%)	0 (0)	362 (100)	< 0.001*

*statistically significant

*AKI* acute kidney injury, *APACHE IV* Acute Physiology and Chronic Health Evaluation, *BMI* body mass index, *GI* gastrointestinal, *ICU* intensive care unit, *IQR* interquartile range, *LOS* length of stay, *pts.* points, *SOFA* sequential organ failure assessment, *UTI* urinary tract infection

### Delta-lactate in severe hyperlactatemia

**Fig. 1:**
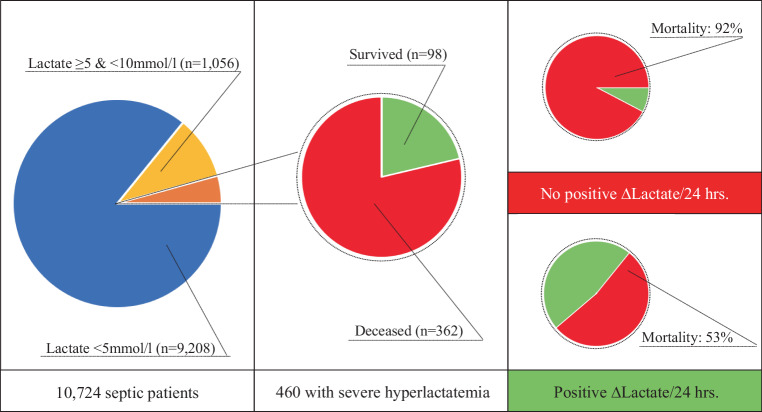
Of a total of 10,724 septic patients from the eICU Collaborative Research Database, 9208 individuals (85.9%) had low (serum lactate < 5 mmol/l), 1056 patients (9.8%) moderate (serum lactate ≥ 5 and < 10 mmol/l) and 460 participants (4.3%) severe hyperlactatemia (≥ 10 mmol/l). Of all patients with severe hyperlactatemia, 98 (21%) survived and 362 (79%) died. Patients from the severe hyperlactatemia group with a positive delta lactate at 24 h had significantly lower in-hospital mortality (53%) than patients without a positive 24‑h delta lactate (92%, *p* < 0.001)

As for individuals in group 3 with a positive delta-lactate, patients were again younger (median age 61 vs. 67 years; *p* = 0.032) and had lower SOFA (13 vs. 14 points; *p* = 0.037) but not APACHE IV scores (110 vs. 114 points; *p* = 0.18) compared to patients without a positive delta-lactate. With regards to the outcome analysis, patients with a positive delta-lactate showed significantly higher survival rates (ICU mortality 42 vs. 85%, *p* < 0.001; hospital mortality 53 vs. 92%, *p* < 0.001), as well as longer ICU stays (134 vs. 47 h; *p* < 0.001), lower rates of AKI (16 vs. 34%; *p* = 0.006) but no differences with respect to mechanical ventilation or vasopressor use. A detailed overview is provided in Table 3. An overview of the results is given in the form of a graphical abstract in Fig. 1.

**Table 3:** Positiveversus no delta-lactate in patients severe hyperlactatemia

Characteristic	Positive ∆ Lactate “Yes”	Positive ∆ Lactate “No”	*p*-value
	*n* *=* *119*	*n* *=* *65*	
Age (years)—median (IQR)	61 (53–73)	67 (57–74)	0.032*
Age > 65 years—*n* (%)	46 (39)	35 (54)	0.047*
Male sex—*n* (%)	54 (45)	33 (51)	0.48
BMI (kg/m^2^)—median (IQR)	28 (23–33)	27 (23–31)	0.62
SOFA score (pts.)—median (IQR)	13 (10–15)	14 (12–16)	0.037*
APACHE IV score (pts.)—median (IQR)	110 (85–132)	114 (96–135)	0.18
Septic shock—*n* (%)	80 (68)	41 (63)	0.52
*Ethnicity*	–	–	0.50
African American—*n* (%)	20 (17)	9 (14)	–
Asian—*n* (%)	1 (1)	2 (3)	–
Caucasian—*n* (%)	81 (69)	46 (72)	–
Hispanic—*n* (%)	6 (5)	5 (8)	–
Native American—*n* (%)	3 (3)	0 (0)	–
Other/Unknown—*n* (%)	7 (6)	2 (3)	–
*Laboratory values*	–	–	–
Maximum lactate day 1 (mmol/L)—median (IQR)	12.9 (11.0–15.4)	12.7 (11.4–14.5)	0.84
Baseline lactate > 2 mmol/L—*n* (%)	118 (99)	63 (97)	0.25
Hemoglobin (g/dL)—median (IQR)	9.7 (8.2–11.2)	10.2 (8.7–11.8)	0.10
White blood count (G/L)—median (IQR)	14.8 (8.4–22.8)	13.4 (4.1–20.0)	0.12
Lymphocyte count (G/L)—median (IQR)	0.7 (0.4–1.0)	0.7 (0.3–1.3)	0.91
Platelet count (G/L)—median (IQR)	143 (78–240)	99 (58–175)	0.007*
Serum creatinine (mg/dL)—median (IQR)	2.4 (1.7–4.2)	2.8 (2.1–3.9)	0.44
*Admission diagnosis*	–	–	0.94
Pulmonary focus—*n* (%)	31 (26)	17 (26)	–
GI focus—*n* (%)	27 (23)	17 (26)	–
Renal focus/UTI—*n* (%)	20 (17)	9 (14)	–
Cutaneous/soft tissue focus—*n* (%)	9 (8)	3 (5)	–
Other focus—*n* (%)	10 (8)	5 (8)	–
Unknown focus—*n* (%)	22 (18)	14 (22)	–
*Intensive care measures*	–	–	–
Mechanical ventilation—*n* (%)	74 (62)	36 (55)	0.37
Vasopressor use—*n* (%)	81 (68)	42 (65)	0.63
AK—*n* (%)	18 (16)	21 (34)	0.006*
LOS (hours)—median (IQR)	134 (59–249)	47 (37–92)	< 0.001*
LOS > 7 days—*n* (%)	48 (40)	11 (17)	0.001*
ICU mortality—*n* (%)	50 (42)	55 (85)	< 0.001
Hospital mortality—*n* (%)	63 (53)	60 (92)	< 0.001*

*statistically significant

*AKI* acute kidney injury, *APACHE IV* Acute Physiology And Chronic Health Evaluation, *BMI* body mass index, *GI* gastro-intestinal, *ICU* intensive care unit, *IQR* interquartile range, *LOS* length of stay, *pts.* points, *SOFA* sequential organ failure assessment, *UTI* urinary tract infection

### Sensitivity analysis

Sensitivity of severe hyperlactatemia for hospital mortality was 18%, specificity 99%, the LR+ was 15.7, the LR− was 0.83. Severe hyperlactatemia was associated with increased odds for hospital mortality (aOR 18.7; 95% CI 14.5–24.2; *p* < 0.001) and remained so after adjustment for APACHE-IV (aOR 7.5 95% CI 5.7–9.9; *p* < 0.001).

## Discussion

In our work on 10,724 septic patients, we observed excessive mortality in the presence of severe hyperlactatemia (≥ 10 mmol/l) in the first 24 h. Furthermore, a high specificity (99%) was observed, which means that only very few patients are attributed an erroneously poor outcome with this finding. On the other hand, the overall high mortality rates in sepsis (even in absence of severe hyperlactatemia) are explanatory for the low sensitivity. In addition, we could show that a positive delta-lactate in the first 24 h is a valid prognostic indicator for a better outcome.

Nowadays, a shift of paradigm away from the hypoxic genesis, to lactate as a source of energy in shock and indicator of stress at the cellular level even in normoxemic states, can be observed [12]. Multiple studies found various constellations of serum lactate to be associated with mortality in all different kinds of critically ill patients in the past which makes its value in critical care undisputed [13]. Whether changes over time, absolute values, and/or specific cut-offs are better predictors will not be the subject of the following discussion and content of this paper. We would like to address the following question “What distinguishes patients with severe hyperlactatemia from others and what are its therapeutic implications?”

To briefly review the existing literature, two large-scale studies on general ICU populations have investigated the sequelae of severe hyperlactatemia in the past. Haas et al. evaluated severe hyperlactatemia and lactate kinetics (first 12 h) in a large patient collective of 14,040 unselected ICU patients. They also found high ICU mortality rates (78.2%) in patients with severe hyperlactatemia (≥ 10 mmol/l), and individuals with persistent hyperlactatemia had even worse outcomes (96.6% ICU mortality if 12‑h lactate clearance < 32.8%) [8]. Gharipour et al. also investigated whether severe hyperlactatemia and 12‑h lactate clearance were good prognostic parameters in their analysis on 23,598 general ICU patients of the MIMIC III database. They also found a high ICU mortality in patients with a maximum lactate ≥ 10 mmol/l (65%), as well as in individuals with a lactate “clearance” < 0% (88.7%) [14]. Masyuk et al. performed another interesting study when they assessed delta-lactate (between days 1 and 2) in 2191 ICU patients. They found a delta-lactate ≤ 19% to be associated not only with lower in-hospital but even long-term survival (up to 9 years of follow-up) [15]. To our knowledge, our study is the largest outcome analysis of purely septic patients with severe hyperlactatemia (≥ 10 mmol/l).

Regarding the observed age-related differences in our study, with younger patients in the severe hyperlactatemia group, possibly age-adjusted lactate cut-offs could be used in the future. This finding was already observed in the past, but no general recommendations for age-adjusted lactate thresholds exist to date [16, 17]. Cannon et al. in their study on 8796 emergency department patients were able to show higher mean lactate levels in younger nonsurvivors compared to older patients and also suggested to adjust conventional lactate thresholds for age [18]. Whether a causal relationship exists in our study or if younger critically ill patients were admitted more often to ICUs compared to older individuals with the same clinical constellation cannot be differentiated from our set of data. Likewise, our study shows differences with respect to ethnicity, which we cannot explain with certainty, although this finding has already been shown in other publications [17, 19]. It may be possible to adapt lactate thresholds to ethnicity analogously to the estimated glomerular fraction rate (e.g. CKD-EPI formula), although this would require large-scale, age-stratified studies with healthy and diseased patients. Nevertheless, it is probably not sufficient to link measurements to ethnicity alone, without including socioeconomic and environmental data to avoid bias [20, 21]. It should also be noted that our study did not show any differences in terms of ethnicity, with regards to actual mortality or delta-lactate in the severe hyperlactatemia group.

The differences in terms of higher APACHE-IV and SOFA scores, as well as a higher incidence of septic shock in the severe hyperlactatemia group is not surprising, as lactate values correlate with disease severity and therefore sicker patients usually evidence higher lactate levels [12, 22]. Also, higher creatinine levels are to be expected in the sicker patient population of individuals with severe hyperlactatemia, as AKI is common in septic patients and sicker patients generally have higher rates of renal failure [23]. Whether patients with worse renal function due to AKI metabolize lactate to a lesser extent cannot be answered in this way, nor does our work show a statistically significant correlation between serum creatinine, severe hyperlactatemia and survival or positive delta-lactate. Also, lactate per se is not the root of the problem, only the lack of lactate utilization in the presence of AKI could indicate metabolic failure, as the kidneys are responsible for about 50% of the body’s lactate metabolism [13].

Hematologic alterations in septic patients are common [24]. In our study, we were able to show significant differences for hemoglobin values, thrombocyte and lymphocyte counts, as well as a trend in absolute leukocyte counts. The frequency of anemia in sepsis has already been reported in various studies, although it is not a strong risk predictor by itself [24]. White blood cell counts are also frequently altered in septic patients, but are considered a nonspecific marker of disease severity [25]. Of interest are changes in leukocyte subpopulations; for example, relative lymphopenia in particular is often observed in hyperinflammation, whereas absolute counts of lymphocytes are often elevated as various lymphocyte subpopulations like natural killer cells or regulatory T‑cells are upregulated in general inflammation [26]. In our study, a particularly strong difference is seen with respect to platelet counts between the different lactate groups. Thus, the lowest platelet counts were observed in the severe hyperlactatemia group, and also here, the lowest counts were seen in nonsurvivors. Furthermore, patients lacking a positive delta-lactate also had lower platelet counts than their counterparts. Thrombocytopenia in sepsis is common, because sepsis is often associated with excessively activated coagulation and platelets also play an important role in the general immune response [27]. In particular, septic patients with a lack of resolution of thrombocytopenia were shown to suffer from a higher mortality [28]. This is interesting, as in our study patients without a positive delta-lactate also had significantly lower platelet counts. A combined model of positive delta-lactate with resolution of thrombocytopenia would be interesting here to map the lack of improvement of the generalized disturbances by combining a volatile marker (delta-lactate) with a more laggard parameter (resolution of thrombocytopenia).

Regarding the focus of infection, significantly more patients with unknown or abdominal focus were observed in the severe hyperlactatemia group, whereas pulmonary or urologic foci were most frequently causative in patients with lactate < 5 mmol/l. There is no clear explanation for this finding, nor is much data available. Kang et al. showed a similar finding in their work, but large studies focusing on this topic do not exist [29]. Also, the usefulness of such studies is questionable, as lactate per se is not indicative of the underlying etiology. Nevertheless, it supports the relevance of hyperlactatemia under normoxemia, as one would expect hypoxic conditions especially in pulmonary disease with respiratory compromise.

If one looks at the secondary endpoints, patients with severe hyperlactatemia are clinically sicker patients, as they need more vasopressors and mechanical ventilation and have a worse outcome. We interpret our findings as one should see lactate and sepsis, namely as deranged pathophysiology on the tissue level. Lactate is neither the cause of the problem, nor the problem itself, sepsis is a dysregulated state with increased generation of lactate by hypermetabolism on the one hand and underutilization as an expression of metabolic arrest on the other hand. Lactate is an indicator of stress, but also an important energy source in the shock state. Future studies should address why it cannot be utilized in some patients, not how to degrade it.

### Limitations

In addition to the retrospective nature of this study, one limitation is that patients were not classified by the recent “Sepsis-3” definition but according to APACHE IV. Nevertheless, we think that due to the large number of patients and, moreover, uniform classification, the scientific value of our work is not compromised. Also, lactate values from the second day after admission are missing in some patients, which is why the delta-lactate could only be calculated for part of the patients. Furthermore, long-term outcome data are not available, although we were able to obtain at least hospital mortality in addition to ICU mortality.

## Conclusion

Our large-scale analysis on over 10,000 patients with sepsis shows high mortality in patients with severe hyperlactatemia (≥ 10 mmol/l) in the first 24 h, especially when no trend towards sufficient utilization is discernible. Due to the high specificity, such a finding should be included in treatment decisions, especially if other clinical factors are present that speak against further treatment escalation. Future studies should particularly address the underlying cause of hyperlactatemia and underutilization of lactate in sepsis, to consider possible therapeutic approaches.

## References

[1] Singer M, Deutschman CS, Seymour CW, Shankar-Hari M, Annane D, Bauer M (2016). The third international consensus definitions for sepsis and septic shock (sepsis-3). JAMA.

[2] Vincent J-L, De Backer D (2013). Circulatory shock. N Engl J Med.

[3] Vincent J-L, Marshall JC, Namendys-Silva SA, François B, Martin-Loeches I, Lipman J (2014). Assessment of the worldwide burden of critical illness: the intensive care over nations (ICON) audit. Lancet Respir Med.

[4] van Vught LA, Klein Klouwenberg PMC, Spitoni C, Scicluna BP, Wiewel MA, Horn J (2016). Incidence, risk factors, and attributable mortality of secondary infections in the intensive care unit after admission for sepsis. JAMA.

[5] Prescott HC, Angus DC (2018). Enhancing recovery from sepsis: a review. JAMA.

[6] Engel FL, Winton MG, Long CN (1943). Biochemical studies on shock: I. The metabolism of amino acids and carbohydrate during hemorrhagic shock in the rat. J Exp Med.

[7] Levy B (2006). Lactate and shock state: the metabolic view. Curr Opin Crit Care.

[8] Haas SA, Lange T, Saugel B, Petzoldt M, Fuhrmann V, Metschke M (2016). Severe hyperlactatemia, lactate clearance and mortality in unselected critically ill patients. Intensive Care Med.

[9] Pollard TJ, Johnson AEW, Raffa JD, Celi LA, Mark RG, Badawi O (2018). The eICU Collaborative Research Database, a freely available multi-center database for critical care research. Sci Data.

[10] O’Halloran HM, Kwong K, Veldhoen RA, Maslove DM (2020). Characterizing the patients, hospitals, and data quality of the eICU collaborative research database. Crit Care Med.

[11] Khwaja A (2012). KDIGO clinical practice guidelines for acute kidney injury. Nephron Clin Pract.

[12] Garcia-Alvarez M, Marik P, Bellomo R (2014). Sepsis-associated hyperlactatemia. Crit Care.

[13] Hernandez G, Bellomo R, Bakker J (2019). The ten pitfalls of lactate clearance in sepsis. Intensive Care Med.

[14] Gharipour A, Razavi R, Gharipour M, Modarres R, Nezafati P, Mirkheshti N (2021). The incidence and outcome of severe hyperlactatemia in critically ill patients. Intern Emerg Med.

[15] Masyuk M, Wernly B, Lichtenauer M, Franz M, Kabisch B, Muessig JM (2019). Prognostic relevance of serum lactate kinetics in critically ill patients. Intensive Care Med.

[16] Oh DH, Kim MH, Jeong WY, Kim YC, Kim EJ, Song JE (2019). Risk factors for mortality in patients with low lactate level and septic shock. J Microbiol Immunol Infect.

[17] Liu Z, Meng Z, Li Y, Zhao J, Wu S, Gou S (2019). Prognostic accuracy of the serum lactate level, the SOFA score and the qSOFA score for mortality among adults with Sepsis. Scand J Trauma Resusc Emerg Med.

[18] Cannon C, Miller R, Grow K, Purcell S, Nazir N (2020). Age-adjusted and expanded lactate thresholds as predictors of all-cause mortality in the emergency department. WestJEM.

[19] Mikkelsen ME, Miltiades AN, Gaieski DF, Goyal M, Fuchs BD, Shah CV (2009). Serum lactate is associated with mortality in severe sepsis independent of organ failure and shock. Crit Care Med.

[20] Galiatsatos P, Brigham EP, Pietri J, Littleton K, Hwang S, Grant MJ (2018). The effect of community socioeconomic status on sepsis-attributable mortality. J Crit Care.

[21] Mayr FB, Yende S, Linde-Zwirble WT, Peck-Palmer OM, Barnato AE, Weissfeld LA (2010). Infection rate and acute organ dysfunction risk as explanations for racial differences in severe sepsis. JAMA.

[22] Chambers KA, Park AY, Banuelos RC, Darger BF, Akkanti BH, Macaluso A (2018). Outcomes of severe sepsis and septic shock patients after stratification by initial lactate value. World J Emerg Med.

[23] Zarjou A, Agarwal A (2011). Sepsis and acute kidney injury. J Am Soc Nephrol.

[24] Aird WC (2003). The hematologic system as a marker of organ dysfunction in sepsis. Mayo Clin Proc.

[25] Farkas JD (2020). The complete blood count to diagnose septic shock. J Thorac Dis.

[26] Tschaikowsky K, Hedwig-Geissing M, Schiele A, Bremer F, Schywalsky M, Schüttler J (2002). Coincidence of pro- and anti-inflammatory responses in the early phase of severe sepsis: Longitudinal study of mononuclear histocompatibility leukocyte antigen-DR expression, procalcitonin, C-reactive protein, and changes in T-cell subsets in septic and postoperative patients. Crit Care Med.

[27] Assinger A, Schrottmaier WC, Salzmann M, Rayes J (2019). Platelets in sepsis: an update on experimental models and clinical data. Front Immunol.

[28] Venkata C, Kashyap R, Farmer JC, Afessa B (2013). Thrombocytopenia in adult patients with sepsis: incidence, risk factors, and its association with clinical outcome. J Intensive Care.

[29] Kang YR (2011). Initial Lactate Level and Mortality in Septic Shock Patients with Hepatic Dysfunction. Anaesth Intensive Care.

